# Comprehensive measurement of UVB-induced non-melanoma skin cancer burden in mice using photographic images as a substitute for the caliper method

**DOI:** 10.1371/journal.pone.0171875

**Published:** 2017-02-10

**Authors:** Marc Bazin, Nupur K. Purohit, Girish M. Shah

**Affiliations:** Laboratory for Skin Cancer Research, CHU-Q (CHUL) Quebec University Hospital Research Centre, Laval University, Québec City, Québec, Canada; Ohio State University Wexner Medical Center, UNITED STATES

## Abstract

The vernier caliper has been used as a gold standard to measure the length, width and height of skin tumors to calculate their total area and volume. It is a simple method for collecting data on a few tumors at a time, but becomes tedious, time-consuming and stressful for the animals and the operator when used for measuring multiple tumors in a large number of animals in protocols such as UVB-induced non-melanoma skin cancer (NMSC) in SKH-1 mice. Here, we show that photographic images of these mice taken within a few minutes under optimized conditions can be subjected to computerized analyses to determine tumor volume and area as accurately and precisely as the caliper method. Unlike the caliper method, the photographic method also records the incidence and multiplicity of tumors, thus permitting comprehensive measurement of tumor burden in the animal. The simplicity and ease of this method will permit more frequent monitoring of tumor burden in long protocols, resulting in the creation of additional data about dynamic changes in progression of cancer or the efficacy of therapeutic intervention. The photographic method can broadly substitute the caliper method for quantifying other skin pathologies.

## Introduction

In mouse models that examine the causes and cures of chronic ultraviolet B (UVB)-induced non-melanoma skin cancers (NMSC), the tumor burden is quantified as the incidence (proportion of mice with or without tumor), multiplicity (number of tumors), tumor area and tumor volume [1–7]. While the first two parameters are visually noted and recorded, the last two parameters are measured using the vernier caliper, which is a simple yet accurate instrument to manually measure the length, width and height of tumors. The use of digital calipers with 0.01 mm accuracy is perfect for collecting data for one or two tumors per animal and for few animals at a time. However, it becomes time-consuming and challenging for measuring dimensions of hundreds of tumors. For example, the chronic UVB-irradiated SKH-1 albino hairless mice develop numerous papillomas, keratocanthomas, carcinomas-in-situ and carcinomas over a period of 10–40 weeks; and caliper method would take 5–30 min per mouse and several days for each cycle of measurement for a large number of mice. Moreover, the repetitive and tedious nature of this work and stress-related movements of forcibly restrained mice increase the chances of errors in caliper measurement. Therefore, although frequent measurement of tumor dimensions throughout the NMSC protocol would provide valuable information about the dynamic state of the disease, few studies report weekly or biweekly measurement of tumor volume [2, 3], while most studies measure tumor volume only at the end of the protocol [4–7]. Some studies do not measure smaller tumors [1, 8] or height of the tumors [1, 9], while others do not report any measurement of tumor size but only the incidence [10]. All these studies produced valid data, but availability of a convenient yet accurate and reproducible alternative to caliper method would permit frequent measurement of tumor area and volume revealing more information about the disease.

In subcutaneous tumor models, the magnetic resonance imaging [11] or ultrasound imaging [12] were shown to be more accurate in measuring tumor volume than caliper method. However, these techniques would be expensive, require each mouse to be anesthetised and impractical for NMSC protocol with large number of mice. In studies related to healing of skin wounds, the area measurements are made with a foot-ruler or tracing of wound shapes on transparent sheets with a grid and scale-pattern, or by spectrophotogrammetry or spectophotography methods that use multiple cameras or video-camera with customized software in a special equipment [13–16]. For NMSC measurement, the transparency method would not save much time over caliper method, and photo or videography-based methods would be costly and perhaps even more time consuming than caliper method. Nonetheless, the photographic methods were more accurate in measurement of wound area, and an earlier NMSC study reported use of photography to determine tumor area [9], although the technique was not validated with caliper method, and it did not report tumor volume possibly because height could not be measured in their technique. Here, we provide detailed description of a fully validated simple photography method that allows measurement of area and volume of skin tumors as accurately as the caliper method, while avoiding potentially error-inducing limitations of the caliper method. This method also permits measurement of the incidence and multiplicity of tumors, thus providing a comprehensive method to measure tumor burden in UVB-induced NMSC protocols, and could broadly substitute caliper method for quantifying other skin pathologies.

## Materials and methods

### Chronic UVB-induced NMSC in SKH-1 hairless mice

All animal studies were approved by the Animal Protection Committee of Laval University and were conducted by the personnel who were trained and certified for animal work. The 5-week old SKH-1 albino hairless mice obtained from Charles River Canada were irradiated thrice a week for 20 weeks at 800 J/m^2^ UVB (280–320 nm). Five weeks after last irradiation, mice were sacrificed under anaesthesia with isoflurane followed by exposure to CO_2_ and cervical dislocation. The unrestrained mice were irradiated in an open cage placed in a Spectrolinker XL-1500 (Spectronics Corp.) equipped with six 15W UVB tube-lights, which delivered 800 J/m^2^ within 2 to 3 min at a flux of 5.9 J/m^2^/sec. During irradiation, the cages were covered from the top with a Kodacel filter to remove the contaminating UVC radiations (230–280 nm) [17]. The UVA wavelengths (320–400 nm) accounted for 20% of the energy, as measured by UVX radiometer (UVP Inc.) equipped with UV-A, B and C-specific probes. Mice were monitored weekly for tumor burden, as described below.

### Caliper method to measure tumor dimensions

The tumors were visible from 12–15 weeks and all data presented in this study are from measurements between 20 to 25 weeks. The pre-neoplastic foci that persisted and progressed over time to form proper tumors were taken into account for the final tumor burden. In contrast, very few cysts that appeared on the skin were identified and excluded from data sets as they either did not change in appearance or disappeared in few weeks. Mice were held in hand for measuring all three dimensions of each tumor using the digital caliper with 0.01 mm precision (Mitutoyo). The length was measured along its longest linear dimension on the skin and the width was measured along the axis perpendicular to the length axis. The height was measured at the tallest point of the tumor. For the length and width that are measured along the flat skin, we could maintain the accuracy of 0.1 mm. However, the heights below 0.5 mm posed practical difficulties in judging accurate placement of the caliper jaws from skin to the top of the tumor; therefore using visual cues and caliper readout, the near-flat tumors were assigned 0.1 mm height and progressively raised tumors were assigned 0.25 or 0.5 mm heights. All heights above 0.5 mm were actual caliper readouts. Assuming hemi-ellipsoidal shape of the tumors, the tumor burden was determined as the area $\left(\frac{\pi }{4}\times length\times width\right)$ and volume $\left(\frac{\pi }{6}\times length\times width\times height\right)$ [6]. The statistical difference in values by two methods was derived using Origin Pro 2015 with the Wilcoxon signed rank test.

### Photographic method to measure tumor dimensions

#### Photography set-up for mice in laminar hood

**Fig 1:**
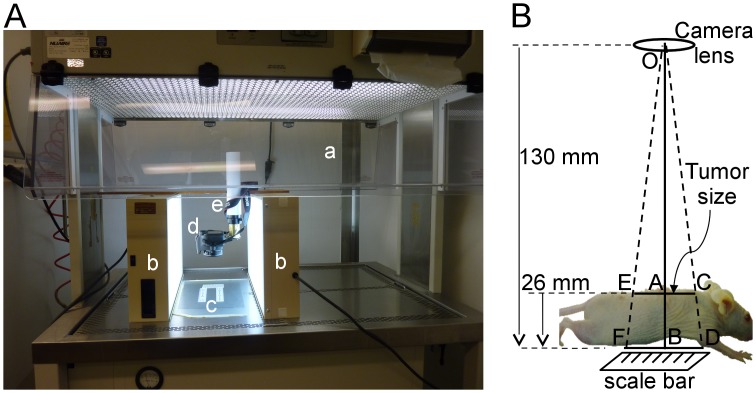
The optimized photography set-up for imaging mice. **(A)** The photography set-up. In a laminar flow cabinet with dual opening (a), illuminated by two additional fluorescent lights on the side (b), mice were placed one at a time on a frosted glass plate with a broad-grid fabric tape that was positioned above black paper and two scale bars (c). The remote shutter controlled camera (d) was positioned 130 mm above the glass plate on a telescopic support (e). **(B)** Representation of the imaging set-up with distances. The camera lens (O) was placed 130 mm above the glass plate, which resulted in a height difference of 26 mm between the scale bar below the glass plate (BD) and tumor (AC) on the dorsal skin of the mouse from the camera lens. The length or width dimension of the tumor (AC) was calculated using Thales’ theorem to determine the scale factor of OA/OB = 0.8 = AC/BD. Since tumors were distributed broadly over the dorsal skin of the mouse, each tumor was carefully calibrated against the nearest portion of the scale bar for better accuracy of measurement. For example, the tumors in EC region of the skin were calibrated against FD segment of the scale.

The measurement of tumor dimensions with photographs requires a set-up with known distances from the camera to the mouse skin and reference scale-bars, as well as an environment that keeps the mouse calm for few minutes to take the pictures of the tumors (Fig 1A). We used a laminar flow cabinet open on both sides for the ease of operations by two persons standing on either side of the cabinet. The cabinet was illuminated with diffused light from a top fluorescent lamp and two 5000 K fluorescent tubes from the sides. We designed a stand that permitted the camera to be steadily positioned so that the lens of the camera was exactly 130 mm above the frosted glass plate that was placed in the cabinet on top of a black paper and two rulers that would flank the mouse in the picture (Fig 1B). Since mouse becomes restless on the glass that does not provide any grip for their paws, we affixed a broad grid fabric tape on which mouse could obtain a secure grip and remain steady for the duration of the photography session. We used a reflex Canon EOS Rebel T5 camera equipped with a Canon EFS 24 mm lens having a fixed focal length and took all the pictures without flash. To avoid shaking the camera or alarming the mouse with hand movement, the pictures were taken using a commercially available remote shutter control cable for the camera.

#### Photography of mice

**Fig 2:**
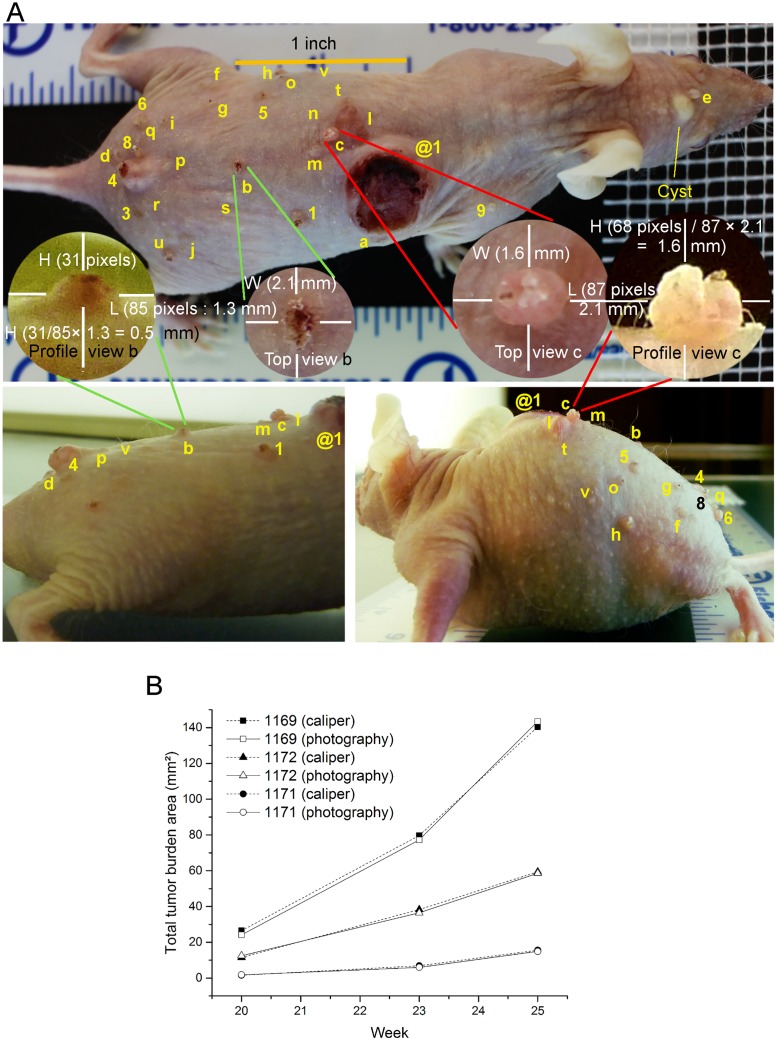
Measurement of tumor dimensions and total tumor burden in mice by two methods. **(A**) Top and profile view pictures of a mouse with multiple UVB-induced skin cancers for photographic method of measuring tumor dimensions. The top views of the mouse with scale bars (top image) were used for detecting tumors (incidence) and for counting the tumors to determine the multiplicity. The magnified images of individual tumors from top view, as shown here for tumors # b and c, were used for measuring the length and width of tumors. The profile pictures of the mouse (bottom images) allowed measurement of heights of all tumors, as shown here for encircled magnified images of these two tumors in different profile images. The calibration of pixel to distance in profile pictures was achieved by comparing either the length or width dimension of a given tumor in both top and profile magnified images. The newly calibrated pixel distances in profile picture now allowed accurate measurement of height of this tumor in profile images. The cysts such as the one seen between eyes were identified as described in the caliper method were excluded in tumor counts. **(B)** Comparison of time-course of increasing total tumor burden (area) in mice as measured by the caliper and photography methods. The total tumor burden (area) in three different mice was monitored from 20 to 25 weeks of the protocol by caliper and photography methods.

The mouse was gently placed over the glass plate and the tail was slightly tugged, which resulted in a relatively stable posture of the mouse with a straight spine and nearly flat dorsal skin containing almost all the tumors. In this situation, the tumors on back of mouse were at an average distance of 104 mm from the camera, whereas the reference scales below the glass plate were at 130 mm from the camera; and these two values were taken into account for calculation of scale factor in images (Fig 1B). Within 30 seconds, 3–5 top view pictures were taken without flashlight that would allow measurement of length and width (Fig 2A, top image). The camera was then removed from the stand to rapidly take several profile pictures of the mouse within 2 min from various angles that would allow measurement of heights of tumors (Fig 2A, bottom images). For profile pictures, it was impractical to use vertical scales or keep a fixed distance between camera and the tumors, as this would unduly prolong picture session. Moreover, the distances in profile pictures could be calibrated against the top picture, as described below.

#### Measurement of the length, width and height of tumors from the top and profile pictures

We used freely available AxioVision SE64-4.9.1 image analysis software (Zeiss) to process the pictures for determining the tumor dimensions, although any other image analyses software that allows correlation of pixel distances in the pictures with actual size based on a scale bar in the same picture is suitable for this analysis. For each mouse, the top picture with scale bars was analyzed first to establish the correlation of pixel to distance in mm using Scaling wizard function in the “Measure/Scaling” menu of the software (Fig 2A, top image). A scale correction factor of 0.8 was applied to account for the difference in the distance from camera to the tumors (OA = 104 mm) and the nearest scale bar (OB = 130 mm) as per Thales' intercept theorem that OA/OB = AC/BD, where AC is the tumor dimension on the back of mouse and BD is the distance on the scale below the glass plate (Fig 1B). The calibrated pixel distance in the picture allowed measurement of length and width of the tumor using the measure “length” menu of the software (Fig 2A). The photographic method with zooming of pictures on the computer screen allowed better definition of the tumor boundaries and more accurate measurement of distances. For profile pictures, since scale bars and distances were not fixed, we calibrated the pixel distance of either the length or width of the tumor in profile image with the known value of this parameter in the top picture and used the revised scale to measure height of the tumor in the profile picture (Fig 2A, circled images of tumors #b and c in top and profile images). For multiple tumors in close proximity, the calibration made with one tumor could be easily applied to measure dimensions of other tumors of similar size. The area and volume of tumors and statistical analyses were carried out as described for caliper method. The average time for measuring dimensions of a single tumor on the computer by the photography method was about 1–2 min, which is similar to the time required for the caliper method. However, for a mouse with 30 tumors, the actual photography session with mouse would take about 3 min and tumor measurements on computer would be about 30–60 min, whereas in the caliper method, the entire period of 30–60 min would have to be spent with a mouse in hand.

## Results

### Photographic method accurately reflects caliper method for total tumor burden in terms of area and volume per mouse

In a single mouse with 30 tumors of different sizes, the photographic and caliper methods were used for measuring length, width and height of each tumor (Fig 1A). Evidently, the top picture of the mouse enlarged on the computer screen allowed us to note the tumor incidence and identify and count tumor multiplicity. The three dimensions of each tumor were used for determining total tumor volume and area by each method. Both the methods produced statistically similar values for total tumor burden, measured as total area or volume of all the tumors in the mouse (Table 1 and S1 Table). To examine whether photographic method was accurate for all sizes of tumors, we sorted these tumors by volume as small (<2 mm^3^) or large (>2 mm^3^); and observed that the photography and caliper methods once again produced statistically similar total collective volume or area for small or large size tumors (Table 1, bottom panel). We noted that volume of individual smaller tumors often differed significantly between two methods because of differences in height rather than length or width values (S1 Table). This could be attributed to approximation of heights assigned to smaller tumors below 0.5 mm in caliper method (see Materials and Methods), whereas the photographic method using enlarged images allowed precise determination of heights below 0.5 mm. Nonetheless, the global impact of the differences in heights of smaller tumors by two methods was negligible to the total tumor burden on the mouse, which is mostly determined by larger tumors. Thus, photographic method allowed measurement of all four parameters of tumor burden, namely the incidence, multiplicity, area and volume, with last two parameters accurately reflecting the values obtained by the caliper method.

**Table 1:** Comparison of tumor volume and area of 30 tumors by caliper and photographic methods.

	Volume (mm^3^)		Area (mm^2^)	
Tumor ID	Caliper	Photography	Caliper	Photography
v	0.05	0.08	0.82	0.57
o	0.07	0.28	1.04	1.41
j	0.09	0.09	1.38	1.41
3	0.13	0.13	1.88	1.90
r	0.14	0.04	0.85	0.57
m	0.16	0.30	0.95	1.13
9	0.17	0.19	0.50	0.95
8	0.18	0.35	1.10	1.33
i	0.22	0.31	1.30	1.56
n	0.22	0.04	1.33	0.63
p	0.27	0.45	1.63	1.70
g	0.46	0.65	1.38	2.42
e	0.55	0.90	0.64	1.13
t	0.79	0.37	2.36	1.84
a	0.85	1.20	5.09	4.52
8	0.89	1.27	0.64	0.95
l (letter)	0.98	1.35	5.87	6.74
q	1.07	1.33	1.34	1.53
f	1.23	0.80	1.84	2.00
5	1.39	0.66	2.97	2.47
h	1.65	1.42	2.47	2.67
b	1.72	0.71	3.68	2.14
d	1.76	1.68	2.94	2.51
1	1.85	1.91	3.97	4.08
u	2.17	1.76	3.61	3.30
6	2.26	2.40	2.26	2.40
c	2.67	2.81	2.67	2.64
k	3.18	3.36	5.96	5.61
4	35.26	31.76	19.59	17.01
@1(2+7)	276.08	274.66	118.32	121.17
**All tumors** n = 30	338.5	333.3	200.4	200.3
% Difference	1.5%		0.05%	
*P-value*	0.98		0.97	
**Small tumors** n = 24, <2 mm^3^	16.9	16.5	48.0	48.2
% Difference	0.2%		0.4%	
*P-value*	0.23		0.84	
**Large tumors** n = 6, >2 mm^3^	321.6	316.8	152.4	152.1
% Difference	1.5%		0.2%	
*P-value*	0.44		0.69	

The individual volume and area measurements of 30 tumors were derived from the data of all three dimensions of each tumor by both the methods as shown in S1 Table. The small and large tumors (2 mm cut-off by caliper volume) are separated by a double-line. The two methods were compared for measuring the total volume or area for all (n = 30), small (n = 24) or large (n = 6) tumors. The statistical significance of difference between two methods was calculated using Wilcoxon signed rank test.

To confirm our observation that the tumor dimensions measured by photographic method are comparable to those measured by caliper; we examined total tumor area in five additional mice by both the methods. The mice had variable tumor area that ranged from 34 to 200 mm^2^. Once again the photography method produced statistically similar tumor burden data as the caliper method for each mouse (Table 2 and S2 Table).

**Table 2:** Comparison of total tumor burden on 6 mice using photographic and caliper methods.

Mouse #	Number of tumors	Total area by caliper (mm^2^)	Total area from photography (mm^2^)	*P-value*	% Difference
1151	10	34.3	33.0	0.30	3.9%
1154	17	132.3	140.9	1	6.5%
1157	12	43.4	458	0.31	5.4%
1158	22	67.5	68.7	0.36	1.7%
1152	24	69.9	72.5	0.30	3.7%
1680	30	200.4	200.3	0.97	0.05%

Total tumor area for 6 mice was calculated from the length and width of all tumors measured by the caliper and photographic methods. The data for individual tumors in each mouse is shown in S2 Table. The differences between two methods for total tumor burden varied from 0.05–6.5%, but it was statistically not different, as determined by Wilcoxon signed rank test.

Finally, we compared the two methods for measuring the changes in total tumor burden in three mice over a period of six weeks (Fig 2B). Although the total tumor area changed in each mouse by 5–7 fold from 20^th^ to 25^th^ week, both the methods produced similar values for total tumor burden at three different time-points in this period. Thus the photography method produced total tumor burden data similar to the caliper method in multiple mice carrying different sizes of tumors and for assessing growth of the same set of tumors over several weeks in a given mouse.

### Accuracy and precision of photography and caliper methods

Next, we compared the accuracy and precision of photography and caliper method by taking 10 replicate measures of the length of a small tumor (~1 mm) and a medium size tumor (~5 mm) by two methods (Table 3 and S3 Table). The average length from 10 measurements by photography method accurately reflected within 1% the average length derived by the caliper method. The precision of a method refers to the reproducibility of result in repeated measurements, and this is derived from relative standard deviation of replicate values (SD / mean × 100) with lower value showing greater precision. The relative standard deviation values revealed that measurements by both the methods were less precise with smaller tumors as compared to larger tumors. Interestingly, the photography method showed better precision than the caliper method for both 1 and 5 mm dimensions. In summary, if caliper is the gold standard for measurement of tumor dimensions, then photography method accurately and precisely measures the true dimensions of the tumors.

**Table 3:** Accuracy and precision of the photography and caliper methods.

		Small tumor (~1 mm length)		Large tumor (~5 mm length)	
		Caliper	Photography	Caliper	Photography
**Mean Length (mm)**		1.03	1.04	5.36	5.32
**Standard Deviation**		0.16	0.10	0.19	0.10
**Precision**	Relative standard variation	15.9	9.30	3.50	1.90
**Accuracy**	(Photography/Caliper) × 100	100.97		99.30	

The length of a small and medium size tumors were measured 10 times by the same operator under optimum conditions, and averages shown here were derived from the dataset shown in S3 Table. Accuracy of photography method was reflected in providing average length value that is very close to the mean obtained by the caliper method. The precision was derived from the relative standard deviation of repeat measures (SD as % of mean value), which decreases when precision increases.

## Discussion

Our results clearly show that the photographic method is simple, cheap and accurate alternative to the caliper method for measuring tumor burden in terms of area and volume of all sizes of NMSC. In addition, the photographs allow measurement of the incidence as well as multiplicity of tumors, thus providing a comprehensive measure of tumor burden in the animal. Most importantly, our photography method allows measurement of the tumor height and therefore the tumor volume. Since height of NMSC often indicates its degree of progression, measuring the actual height of each tumor has an advantage of integrating the severity of cancer in the volume data. The biggest advantage of photography method is that it splits the total task of caliper method in two distinct components. The first one involves handling of the animals to collect raw data in the form of pictures which are taken within few minutes; while the second task of tumor dimension measurement is handled away from the animals and using computerized methods which can be performed at any other time by the operator who handled the animal or by anybody else with computer skills. Thus, a quick photo session is less stressful to the animal and decreases the operator fatigue. Moreover, the photography method offers more reproducibility and accuracy than the caliper method in volume measurement of even smaller tumors with heights below 0.5 mm. In our experience, the operators who are properly trained in both the methods are able to generate unbiased data for total tumor volume or area from multiple tumors that differ by less than 5% between two methods for each mouse. Thus, while being as accurate as the caliper method, photographic method avoids major error-inducing factors of caliper method even when used for measuring the tumor burden in a large number of animals.

The photography method offers additional advantages, such as reducing the cost associated with using the animal house facilities during long hours of caliper measurement in time-shared laminar flow cabinets. The splitting of tasks for animal handling for picture session and computerized measurement of tumor dimensions permits better work distribution and time-management among personnel with animal and computer skills. Most importantly, the photographs serve as a permanent record of data for regulatory purposes, and could be subjected to repeated analyses for verification or correction of data, as well as for any additional analyses in future. A series of weekly photographs during the protocol could provide useful data for determining the growth rate of tumors from pre-neoplastic foci to full tumors or for regression of tumors in response to therapy. Among few limitations of photography method is the need for a skilled photographer and a well-trained person in computer skills for dimension measurement, as well as the creation of simple photography set-up in the animal facility. In view of its simplicity, the photography method would also be suitable for quantification of skin lesions in the models of melanoma, subcutaneous tumors, wound healing, inflammatory responses and other pathologies of skin.

## Supporting information

S1 Table Three dimensions of 30 individual tumors measured by the caliper and photography methods. (DOCX)

S2 Table The total tumor area (mm^2^) by the caliper (C) and photography (P) methods for six mice bearing 10–30 tumors. (DOCX)

S3 Table Ten replicate measurements of length of a small and a medium size tumor to determine accuracy and precision of the photography and caliper methods. (DOCX)
