# Correction: Comprehensive measurement of UVB-induced non-melanoma skin cancer burden in mice using photographic images as a substitute for the caliper method

**DOI:** 10.1371/journal.pone.0173718

**Published:** 2017-03-06

**Authors:** The PLOS ONE Staff

The captions for Tables 1–3 are incorrectly displayed within the article text rather than with the tables themselves. Please see Tables 1–3 and their affiliated captions below. The publisher apologizes for the error.

**Table 1:** Comparison of tumor volume and area of 30 tumors by caliper and photographic methods. The individual volume and area measurements of 30 tumors were derived from the data of all three dimensions of each tumor by both the methods as shown in S1 Table. The small and large tumors (2 mm cut-off by caliper volume) are separated by a double-line. The two methods were compared for measuring the total volume or area for all (n = 30), small (n = 24) or large (n = 6) tumors. The statistical significance of difference between two methods was calculated using Wilcoxon signed rank test.

	Volume (mm^3^)		Area (mm^2^)	
Tumor ID	Caliper	Photography	Caliper	Photography
v	0.05	0.08	0.82	0.57
o	0.07	0.28	1.04	1.41
j	0.09	0.09	1.38	1.41
3	0.13	0.13	1.88	1.90
r	0.14	0.04	0.85	0.57
m	0.16	0.30	0.95	1.13
9	0.17	0.19	0.50	0.95
8	0.18	0.35	1.10	1.33
i	0.22	0.31	1.30	1.56
n	0.22	0.04	1.33	0.63
p	0.27	0.45	1.63	1.70
g	0.46	0.65	1.38	2.42
e	0.55	0.90	0.64	1.13
t	0.79	0.37	2.36	1.84
a	0.85	1.20	5.09	4.52
8	0.89	1.27	0.64	0.95
l (letter)	0.98	1.35	5.87	6.74
q	1.07	1.33	1.34	1.53
f	1.23	0.80	1.84	2.00
5	1.39	0.66	2.97	2.47
h	1.65	1.42	2.47	2.67
b	1.72	0.71	3.68	2.14
d	1.76	1.68	2.94	2.51
1	1.85	1.91	3.97	4.08
u	2.17	1.76	3.61	3.30
6	2.26	2.40	2.26	2.40
c	2.67	2.81	2.67	2.64
k	3.18	3.36	5.96	5.61
4	35.26	31.76	19.59	17.01
@1(2+7)	276.08	274.66	118.32	121.17
**All tumors** n = 30	338.5	333.3	200.4	200.3
% Difference	1.5%		0.05%	
*P-value*	0.98		0.97	
**Small tumors** n = 24, <2 mm^3^	16.9	16.5	48.0	48.2
% Difference	0.2%		0.4%	
*P-value*	0.23		0.84	
**Large tumors** n = 6, >2 mm^3^	321.6	316.8	152.4	152.1
% Difference	1.5%		0.2%	
*P-value*	0.44		0.69	

**Table 2:** Comparison of total tumor burden on 6 mice using photographic and caliper methods. Total tumor area for 6 mice was calculated from the length and width of all tumors measured by the caliper and photographic methods. The data for individual tumors in each mouse is shown in S2 Table. The differences between two methods for total tumor burden varied from 0.05–6.5%, but it was statistically not different, as determined by Wilcoxon signed rank test.

Mouse #	Number of tumors	Total area by caliper (mm^2^)	Total area from photography (mm^2^)	*P-value*	% Difference
1151	10	34.3	33.0	0.30	3.9%
1154	17	132.3	140.9	1	6.5%
1157	12	43.4	458	0.31	5.4%
1158	22	67.5	68.7	0.36	1.7%
1152	24	69.9	72.5	0.30	3.7%
1680	30	200.4	200.3	0.97	0.05%

**Table 3:** Accuracy and precision of the photography and caliper methods. The length of a small and medium size tumors were measured 10 times by the same operator under optimum conditions, and averages shown here were derived from the dataset shown in S3 Table. Accuracy of photography method was reflected in providing average length value that is very close to the mean obtained by the caliper method. The precision was derived from the relative standard deviation of repeat measures (SD as % of mean value), which decreases when precision increases.

		Small tumor (~1 mm length)		Large tumor (~5 mm length)	
		Caliper	Photography	Caliper	Photography
**Mean Length (mm)**		1.03	1.04	5.36	5.32
**Standard Deviation**		0.16	0.10	0.19	0.10
**Precision**	Relative standard variation	15.9	9.30	3.50	1.90
**Accuracy**	(Photography/Caliper) × 100	100.97		99.30	
