# Supplementary material for: Comprehensive measurement of UVB-induced non-melanoma skin cancer burden in mice using photographic images as a substitute for the caliper method
Source: PLoS One. 2017 Feb 10;12(2):e0171875. doi: 10.1371/journal.pone.0171875 (PMC5302799; doi:10.1371/journal.pone.0171875)
Supplement: S1 Table — (DOCX) [file pone.0171875.s001.docx]

$\boldsymbol{S}\boldsymbol{1 Table. Three dimensions of 30 individual tumors measured by the caliper and}$ **photography methods**

|  |  |  |  |  |  |  |  | **Volume (**$\frac{\boldsymbol{\pi}}{\boldsymbol{6}}\mathcal{\times L\times W\times H)}$ **mm³** | | **Area (**$\frac{\boldsymbol{\pi}}{\boldsymbol{4}}\mathcal{\times L\times W\times H)}$ **mm²** | |
| --- | --- | --- | --- | --- | --- | --- | --- | --- | --- | --- | --- |
|  | **Tumor ID** | **Caliper** | | | **Photography** | | | Volume-Caliper | Volume-Photography | Area-Caliper | Area-Photography |
|  |  | **L** | **W** | **H** | **L** | **W** | **H** | **(Vc)** | **(Vp)** | **(Ac)** | **(Ap)** |
| Less than 2 mm³ | **v** | 1.5 | 0.7 | 0.1 | 1.2 | 0.6 | 0.2 | 0.05 | 0.08 | 0.82 | 0.57 |
|  | **o** | 1.2 | 1.1 | 0.1 | 1.5 | 1.2 | 0.3 | 0.07 | 0.28 | 1.04 | 1.41 |
|  | **j** | 1.6 | 1.1 | 0.1 | 1.5 | 1.2 | 0.1 | 0.09 | 0.09 | 1.38 | 1.41 |
|  | **3** | 2 | 1.2 | 0.1 | 2.2 | 1.1 | 0.1 | 0.13 | 0.13 | 1.88 | 1.90 |
|  | **r** | 1.2 | 0.9 | 0.25 | 0.9 | 0.8 | 0.1 | 0.14 | 0.04 | 0.85 | 0.57 |
|  | **m** | 1.1 | 1.1 | 0.25 | 1.2 | 1.2 | 0.4 | 0.16 | 0.30 | 0.95 | 1.13 |
|  | **9** | 0.8 | 0.8 | 0.5 | 1.1 | 1.1 | 0.3 | 0.17 | 0.19 | 0.50 | 0.95 |
|  | **s** | 1.4 | 1 | 0.25 | 1.3 | 1.3 | 0.4 | 0.18 | 0.35 | 1.10 | 1.33 |
|  | **i** | 1.5 | 1.1 | 0.25 | 1.8 | 1.1 | 0.3 | 0.22 | 0.31 | 1.30 | 1.56 |
|  | **n** | 1.3 | 1.3 | 0.25 | 1 | 0.8 | 0.1 | 0.22 | 0.04 | 1.33 | 0.63 |
|  | **p** | 1.6 | 1.3 | 0.25 | 1.8 | 1.2 | 0.4 | 0.27 | 0.45 | 1.63 | 1.70 |
|  | **g** | 1.6 | 1.1 | 0.5 | 2.2 | 1.4 | 0.4 | 0.46 | 0.65 | 1.38 | 2.42 |
|  | **e** | 0.9 | 0.9 | 1.3 | 1.2 | 1.2 | 1.2 | 0.55 | 0.90 | 0.64 | 1.13 |
|  | **t** | 2 | 1.5 | 0.5 | 1.8 | 1.3 | 0.3 | 0.79 | 0.37 | 2.36 | 1.84 |
|  | **a** | 2.7 | 2.4 | 0.25 | 2.5 | 2.3 | 0.4 | 0.85 | 1.20 | 5.09 | 4.52 |
|  | **8** | 0.9 | 0.9 | 2.1 | 1.1 | 1.1 | 2 | 0.89 | 1.27 | 0.64 | 0.95 |
|  | **l** | 3.4 | 2.2 | 0.25 | 3.3 | 2.6 | 0.3 | 0.98 | 1.35 | 5.87 | 6.74 |
|  | **q** | 1.7 | 1 | 1.2 | 1.5 | 1.3 | 1.3 | 1.07 | 1.33 | 1.34 | 1.53 |
|  | **f** | 1.8 | 1.3 | 1 | 1.7 | 1.5 | 0.6 | 1.23 | 0.80 | 1.84 | 2.00 |
|  | **5** | 2.1 | 1.8 | 0.7 | 2.1 | 1.5 | 0.4 | 1.39 | 0.66 | 2.97 | 2.47 |
|  | **h** | 2.1 | 1.5 | 1 | 2 | 1.7 | 0.8 | 1.65 | 1.42 | 2.47 | 2.67 |
|  | **b** | 2.6 | 1.8 | 0.7 | 2.1 | 1.3 | 0.5 | 1.72 | 0.71 | 3.68 | 2.14 |
|  | **d** | 2.2 | 1.7 | 0.9 | 2 | 1.6 | 1 | 1.76 | 1.68 | 2.94 | 2.51 |
|  | **1** | 2.3 | 2.2 | 0.7 | 2.6 | 2 | 0.7 | 1.85 | 1.91 | 3.97 | 4.08 |
| More than 2 mm³ | **u** | 2.3 | 2 | 0.9 | 2.1 | 2 | 0.8 | 2.17 | 1.76 | 3.30 | 2.17 |
|  | **6** | 1.8 | 1.6 | 1.5 | 1.7 | 1.8 | 1.5 | 2.26 | 2.40 | 2.40 | 2.26 |
|  | **c** | 2 | 1.7 | 1.5 | 2.1 | 1.6 | 1.6 | 2.67 | 2.81 | 2.64 | 2.67 |
|  | **k** | 3.3 | 2.3 | 0.8 | 3.4 | 2.1 | 0.9 | 3.18 | 3.36 | 5.61 | 3.18 |
|  | **4** | 5.8 | 4.3 | 2.7 | 5.7 | 3.8 | 2.8 | 35.26 | 31.76 | 17.01 | 35.26 |
|  | **@1 (2+7)** | 13.1 | 11.5 | 3.5 | 13.3 | 11.6 | 3.4 | 276.08 | 274.66 | 121.17 | 276.08 |
| Volume range (mm³) | | | | | | | **n** | **(Vc)** | **(Vp)** | **(Ac)** | **(Ap)** |
| Sum (All) | | | | | | | **30** | 338.5 | 333.3 | 200.4 | 200.3 |
| Sum (0-2 mm³) | | | | | | | **24** | 16.9 | 16.5 | 48.0 | 48.2 |
| Sum (> 2mm³) | | | | | | | **6** | 321.6 | 316.8 | 152.4 | 152.1 |
