# Supplementary material for: Comprehensive measurement of UVB-induced non-melanoma skin cancer burden in mice using photographic images as a substitute for the caliper method
Source: PLoS One. 2017 Feb 10;12(2):e0171875. doi: 10.1371/journal.pone.0171875 (PMC5302799; doi:10.1371/journal.pone.0171875)
Supplement: S2 Table — (DOCX) [file pone.0171875.s002.docx]

**S2 Table. The total tumor area (mm^2^) by the caliper (C) and photography (P) methods for six mice bearing 10 to 30 tumors**

| **Mouse #** | **1151** | | **1152** | | **1154** | | **1157** | | **1158** | | **1680** | |
| --- | --- | --- | --- | --- | --- | --- | --- | --- | --- | --- | --- | --- |
| **Method** | C | P | C | P | C | P | C | P | C | P | C | P |
| **Tumor #** |  |  |  |  |  |  |  |  |  |  |  |  |
| **1** | 1.4 | 1.5 | 116.5 | 128.8 | 5.9 | 5.6 | 1.8 | 2.7 | 0.8 | 0.8 | 0.5 | 1 |
| **2** | 4.4 | 4.4 | 2.3 | 2.6 | 0.8 | 1 | 1.2 | 1.6 | 1.8 | 1.3 | 0.6 | 1 |
| **3** | 1.2 | 1.3 | 6 | 6.5 | 2.4 | 3.8 | 4 | 4 | 1.3 | 1.4 | 0.6 | 1.1 |
| **4** | 3.2 | 3.1 | 6.3 | 6.4 | 9.3 | 8.6 | 1.8 | 2.1 | 4.6 | 5.5 | 0.8 | 0.6 |
| **5** | 4.8 | 4.4 | 3.8 | 4.5 | 0.8 | 1 | 2.1 | 2.4 | 1.9 | 1.7 | 0.9 | 0.6 |
| **6** | 3 | 1.8 | 3.4 | 3.4 | 1.8 | 3.2 | 2.2 | 2.4 | 1.9 | 2.2 | 1 | 1.1 |
| **7** | 11.8 | 11.2 | 4 | 3.5 | 13.7 | 14 | 1.2 | 1.4 | 2.2 | 3.3 | 1 | 1.4 |
| **8** | 2.3 | 1.8 | 3.4 | 3.4 | 11.2 | 10.5 | 1.7 | 2.3 | 4 | 3.7 | 1.1 | 1.3 |
| **9** | 2 | 1.7 | 3 | 2.4 | 0.9 | 0.8 | 10.7 | 12.5 | 1.2 | 1.9 | 1.3 | 1.6 |
| **10** | 9.6 | 10.8 | 3.4 | 3.2 | 5 | 5.3 | 16.8 | 14.8 | 1.7 | 1.8 | 1.3 | 0.6 |
| **11** |  |  | 1.9 | 2.7 | 2 | 2.7 | 2.1 | 2.1 | 0.5 | 1.3 | 1.3 | 1.5 |
| **12** |  |  | 2.4 | 2.8 | 1.5 | 1.8 | 2.3 | 2 | 0.4 | 1.2 | 1.4 | 2.4 |
| **13** |  |  | 3.2 | 1.7 |  |  | 2.9 | 3.6 | 3.8 | 3.5 | 1.4 | 1.4 |
| **14** |  |  | 1.8 | 1.4 |  |  | 2.8 | 4.8 | 0.8 | 1 | 1.6 | 1.7 |
| **15** |  |  | 3.2 | 3.1 |  |  | 2 | 3.2 | 1.8 | 1.8 | 1.8 | 2 |
| **16** |  |  | 2.1 | 1.6 |  |  | 0.8 | 0.5 | 0.9 | 2 | 1.9 | 1.9 |
| **17** |  |  | 1.8 | 1.4 |  |  | 11.2 | 7.8 | 2.7 | 2.2 | 2.3 | 2.4 |
| **18** |  |  |  |  |  |  | 2.5 | 2.7 | 1 | 0.8 | 2.4 | 1.8 |
| **19** |  |  |  |  |  |  | 4.4 | 3.2 | 4.4 | 4.6 | 2.5 | 2.7 |
| **20** |  |  |  |  |  |  | 8.1 | 6.5 | 5.2 | 5.5 | 2.7 | 2.6 |
| **21** |  |  |  |  |  |  | 1.7 | 2.3 | 40.2 | 39 | 2.9 | 2.5 |
| **22** |  |  |  |  |  |  | 1.7 | 2.6 | 1.8 | 2.1 | 3 | 2.5 |
| **23** |  |  |  |  |  |  |  |  | 2.1 | 1.6 | 3.6 | 3.3 |
| **24** |  |  |  |  |  |  |  |  | 2 | 2.1 | 3.7 | 2.1 |
| **25** |  |  |  |  |  |  |  |  |  |  | 4 | 4.1 |
| **26** |  |  |  |  |  |  |  |  |  |  | 5.1 | 4.5 |
| **27** |  |  |  |  |  |  |  |  |  |  | 5.9 | 6.7 |
| **28** |  |  |  |  |  |  |  |  |  |  | 6 | 5.6 |
| **29** |  |  |  |  |  |  |  |  |  |  | 19.6 | 17 |
| **30** |  |  |  |  |  |  |  |  |  |  | 118.3 | 121.2 |
| Total area | **34.3** | **33.0** | **132.3** | **140.9** | **43.4** | **45.8** | **67.5** | **68.7** | **69.9** | **72.5** | **200.5** | **200.2** |
| Number of tumors | 10 | | 17 | | 12 | | 22 | | 24 | | 30 | |
| Mouse # | Number of tumors | | Total area by caliper (mm²) | | Total area from photography (mm²) | | Wilcoxon test | | \|photography-caliper\|/caliper | | | |
| 1151 | 10 | | 34.3 | | 33 | | 0.3 | | 3.90% | | | |
| 1154 | 12 | | 43.4 | | 45.8 | | 0.31 | | 5.40% | | | |
| 1157 | 22 | | 67.5 | | 68.7 | | 0.36 | | 1.70% | | | |
| 1158 | 24 | | 69.9 | | 72.5 | | 0.3 | | 3.70% | | | |
| 1152 | 17 | | 132.3 | | 140.9 | | 1 | | 6.50% | | | |
| 1680 | 30 | | 200.4 | | 200.3 | | 0.97 | | 0.05% | | | |
