# Supplementary material for: Comprehensive measurement of UVB-induced non-melanoma skin cancer burden in mice using photographic images as a substitute for the caliper method
Source: PLoS One. 2017 Feb 10;12(2):e0171875. doi: 10.1371/journal.pone.0171875 (PMC5302799; doi:10.1371/journal.pone.0171875)
Supplement: S3 Table — (DOCX) [file pone.0171875.s003.docx]

**S3** **Table.** **Ten replicate measurements of length of a small and a medium size tumor to determine** a**ccuracy and precision of the photography and caliper methods.**

| **Supplementary Table S-III** | | |  |
| --- | --- | --- | --- |
|  |  | Small Tumor | Large Tumor |
| Method | Repeat # | Length (mm) | Length (mm) |
|  | 1 | 0.8 | 5.1 |
|  | 2 | 0.9 | 5.1 |
| C | 3 | 0.9 | 5.6 |
| A | 4 | 1.0 | 5.6 |
| L | 5 | 1.0 | 5.5 |
| I | 6 | 1.1 | 5.5 |
| P | 7 | 1.3 | 5.2 |
| E | 8 | 0.9 | 5.3 |
| R | 9 | 1.2 | 5.3 |
|  | 10 | 1.2 | 5.4 |
|  | **Mean** | **1.03** | **5.36** |
|  | Standard deviation | 0.16 | 0.19 |
|  | Relative standard deviation | 15.89 | 3.54 |
| P  H  O  T  O  G  R  A  P  H  Y | 1 | 1.0 | 5.5 |
|  | 2 | 1.1 | 5.4 |
|  | 3 | 1.1 | 5.3 |
|  | 4 | 1.1 | 5.3 |
|  | 5 | 0.9 | 5.3 |
|  | 6 | 0.9 | 5.4 |
|  | 7 | 1.0 | 5.1 |
|  | 8 | 1.1 | 5.3 |
|  | 9 | 1.2 | 5.3 |
|  | 10 | 1.0 | 5.3 |
|  | **Mean** | **1.04** | **5.32** |
|  | Standard deviation | 0.10 | 0.10 |
|  | Relative standard deviation | 9.29 | 1.94 |
